# The Effect of Antihypertensive Medications on Testing for Primary Aldosteronism

**DOI:** 10.3389/fphar.2021.684111

**Published:** 2021-05-13

**Authors:** Piotr Jędrusik, Bartosz Symonides, Jacek Lewandowski, Zbigniew Gaciong

**Affiliations:** Department of Internal Medicine, Hypertension and Vascular Diseases, Medical University of Warsaw, Warsaw, Poland

**Keywords:** primary aldosteronism, renin, aldosterone, aldosterone-to-renin ratio, renin-angiotensin-aldosterone system, antihypertensive drug treatment, mineralocorticoid receptor antagonist

## Abstract

Primary aldosteronism (PA) is a potentially curable form of secondary hypertension caused by excessive renin-independent aldosterone secretion, leading to increased target organ damage and cardiovascular morbidity and mortality. The diagnosis of PA requires measuring renin and aldosterone to calculate the aldosterone-to-renin ratio, followed by confirmatory tests to demonstrate renin-independent aldosterone secretion and/or PA subtype differentiation. Various antihypertensive drug classes interfere with the renin-angiotensin-aldosterone axis and hence evaluation for PA should ideally be performed off-drugs. This is, however, often precluded by the risks related to suboptimal control of blood pressure and serum potassium level in the evaluation period. In the present review, we summarized the evidence regarding the effect of various antihypertensive drug classes on biochemical testing for PA, and critically appraised the issue whether and which antihypertensive medications should be withdrawn or, conversely, might be continued in patients evaluated for PA. The least interfering drugs are calcium antagonists, alpha-blockers, hydralazine, and possibly moxonidine. If necessary, the testing may also be attempted during treatment with beta-blockers, angiotensin-converting enzyme inhibitors, and angiotensin receptor blockers but renin and aldosterone measurements must be interpreted in the context of known effects of these drugs on these parameters. Views are evolving on the feasibility of testing during treatment with mineralocorticoid receptor antagonists, as these drugs are now increasingly considered acceptable in specific patient subsets, particularly in those with severe hypokalemia and/or poor blood pressure control on alternative treatment.

## Introduction

Primary aldosteronism (PA) is a potentially curable form of secondary hypertension caused by excessive renin-independent aldosterone secretion. Patients with PA have a higher risk of target organ damage and cardiovascular morbidity and mortality compared to matched subjects with essential hypertension and the same blood pressure values (Monticone et al., 2018). Excess aldosterone is associated with hypertension, hypokalemia, renal toxicity, metabolic disorders, and cardiovascular damage. The most predominant causes of PA include bilateral adrenal hyperplasia (60–70%) and unilateral aldosterone-producing adenoma (APA). There are also less frequent forms like unilateral adrenal hyperplasia, genetically determined familial hyperaldosteronism and very rare aldosterone-secreting malignancies.

The classical clinical picture of PA includes hypertension and hypokalemia. While hypertension is almost always present in subjects with PA, plasma potassium levels are frequently within normal limits. Data from the recent studies show that less than 50% of patients present with hypokalemia, more often subjects with APA than with bilateral hyperplasia. The blood pressure in PA is usually substantially elevated, in particular in patients with APA. In a retrospective analysis of patients with resistant hypertension, 182 (11.3%) of cases of PA were identified (Douma et al., 2008), but PA is rarely found in subjects with malignant hypertension which is generally associated with a high renin activity (Zarifis et al., 1996). The current guidelines recommend to screen for PA in subjects with hypertension and specific clinical conditions like severe or resistant hypertension, hypokalemia (spontaneous or diuretic-induced), adrenal nodule (incidentaloma), sleep apnea, or a family history suggesting a genetic form (Funder et al., 2016).

The initial evaluation should consist of the measurement of plasma aldosterone concentration (PAC) and renin (plasma renin activity [PRA] or direct renin concentration [DRC]) to demonstrate reduced PRA with an inappropriately high level of plasma aldosterone. The aldosterone-to-renin ratio (ARR) has been commonly accepted as a screening test for PA and a value above 20 (if PAC is expressed in ng/dL and PRA is expressed in ng/mL/h), when accompanied by elevated PAC, is considered abnormal, suggesting renin-independent aldosterone excess. To confirm the diagnosis of PA, inappropriate secretion of aldosterone should be demonstrated with maneuvers that reduce renin-dependent aldosterone secretion. Usually, excessive sodium loading, orally or by the intravenous infusion, is used. Less common tests include suppression with synthetic mineralocorticoid fludrocortisone or captopril challenge test. When the diagnosis of PA is confirmed, adrenal computed tomography (CT) is recommended to differentiate between unilateral and bilateral lesions. Many experts recommend that patients with PA referred for surgery should undergo adrenal venous sampling (AVS) before adrenalectomy. This is related to the limitations of adrenal CT which frequently does not detect small adenomas in a contralateral gland or cannot distinguish between functioning or non-functioning adenomas.

Measurement of ARR in patients with different forms of hypertension does not detect a distinct population of subjects with PA characterized by an elevated ratio. ARR abnormality was defined using different criteria, like the response to surgical treatment, results of suppression tests or comparison to the range seen in normal subjects. In the wide range of hypertensive subjects, ARR represents a continuous variable and cut-off values are defined arbitrarily. This may represent the continuum of renin-independent aldosteronism which also contains subjects with mild-to-moderate hypertension. The systematic review by Kaiser et al. which included 39 studies with over 42,000 patients reported the estimates of prevalence of PA ranging from 3 to 13% in the primary care and 1–30% in specialized centers (Käyser et al., 2016). Recent reports show a high prevalence of unrecognized PA in different groups of subjects with hypertension, even as high as 4% in newly diagnosed patients (Xu et al., 2020) (Table 1).

**TABLE 1:** Recent studies on the prevalence of primary aldosteronism in subjects without convincing indications for screening (modified after (Hundemer et al., 2021)).

Study population (n)	Diagnostic criteria	Country	Prevalence (%)	Authors (year)
Normotension (*n* = 210)	Low renin activity (<1 ng/mL/h) AND positive confirmatory testing (oral salt suppression test)	United States	13.8	Baudrand et al. (2017)
Stage 1 hypertension (*n* = 1,133)	ARR >30 ng/dl/ng/ml/h with aldosterone >10 ng/dl AND positive confirmatory testing (saline infusion or captopril challenge test)	Italy	3.9	Monticone et al. (2017)
Stage 2 hypertension (*n* = 413)			9.7	
Stage 3 hypertension (*n* = 126)			11.8	
Normotension (*n* = 289)	ARR >30 (ng/dl per ng/[ml^−1^x h^−1^] with aldosterone >10 ng/dl AND positive confirmatory testing (saline infusion or captopril challenge test)	United States	11.3	Brown et al. (2020)
Stage 1 hypertension (*n* = 115)			15.7	
Stage 2 hypertension (*n* = 203)			21.6	
Resistant hypertension (*n* = 408)			22.0	
Newly diagnosed hypertension (*n* = 1,020)	ARR >20 ng/mIU AND aldosterone >10 ng/ml AND: captopril challenge test AND/OR saline infusion	China	4.0	Xu et al. (2020)

ARR, aldosterone-to-renin ratio.

**FIGURE 1:**
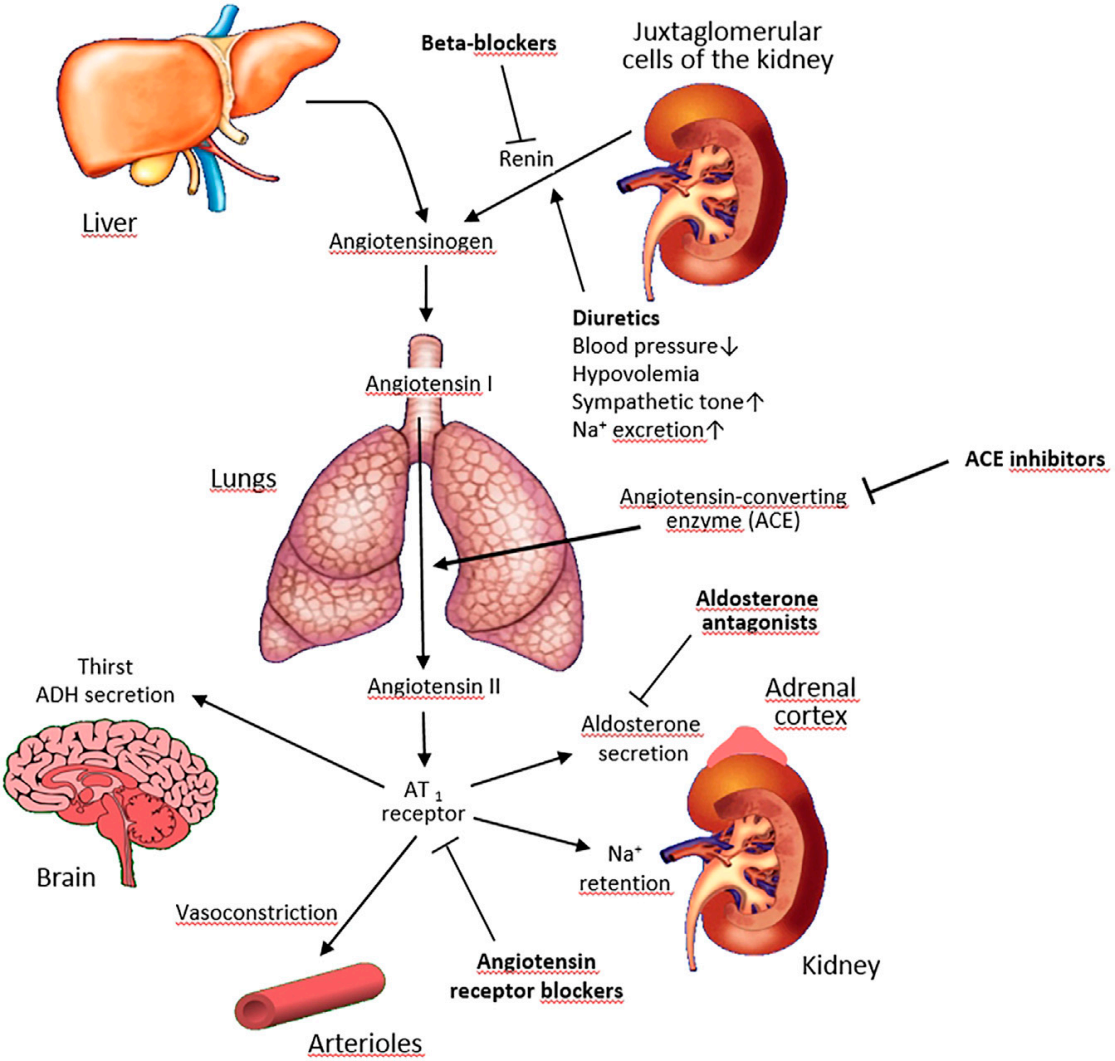
Effects of antihypertensive drugs on the renin-angiotensin-aldosterone system. Pointed arrows indicate stimulation, blunted arrows–inhibition.

To avoid false-positive and false-negative results of biochemical tests, certain conditions must be met. The guidelines recommend to collect blood samples for ARR in the morning after patients have been out of bed for at least 2 h, usually after they have been seated for 5–15 min. The patient should not restrict salt intake before testing and should be potassium-replete. Many drugs interfere with the renin-angiotensin-aldosterone axis (Figure 1) and ideally they should be withdrawn weeks before testing. It can be safely done in subjects with stage 1 hypertension but may create certain risks in other patients.

The need to withdraw some or most antihypertensive medications before testing for PA prolongs the overall diagnostic process, often by many weeks, and limits the feasibility of testing in patients with more severe/resistant hypertension or with a very high cardiovascular risk, e.g., after a recent cardiovascular event, in whom withdrawing all or some medications is deemed unsafe. On the other hand, testing while on antihypertensive medications creates issues with the interpretation of renin and aldosterone measurements for the purpose of diagnosing PA due to the effect of these medications on the biochemical parameters being measured.

Indeed, it has been recently shown in a large cohort study in the United States that testing for PA is rare in patients with resistant hypertension, but if the testing was performed, it was associated with a higher likelihood of initiating mineralocorticoid receptor antagonist (MRA) therapy and better blood pressure control over time (Cohen et al., 2021).

The goal of the present review is to summarize the evidence regarding the effect of various antihypertensive drug classes on biochemical testing for PA, and critically appraise the issue whether and which antihypertensive medications should be withdrawn or, conversely, might be continued in patients evaluated for PA. Based on the available data, we propose an approach to antihypertensive drug regimen modifications when screening for PA.

## Aldosterone, Renin, and Aldosterone-To-Renin Ratio

The aldosterone-to-renin ratio was proposed as a screening test for PA by Hiramatsu et al. (1981). The test was broadly used since then and is recommended for the initial evaluation of patients with suspicion of PA by the current guidelines (Funder et al., 2016; Rossi et al., 2020a). According to the most recent studies, the test has the sensitivity of 100% and the specificity of 89.6% (Pilz et al., 2019). It performs better than the assessment of potassium, aldosterone or renin separately (Rossi et al., 2020a). However, concerns have been recently raised regarding a high intraindividual variability of ARR in the real life conditions and too high thresholds for a positive result of the test in patients with PA (Yozamp et al., 2021). In the past, very good intraindividual variability of the test was reported when performed in standardized conditions (Rossi et al., 2010). The direct measurement of renin concentration (DRC) replaced the assessment of PRA because it is cheaper, quicker and allows to handle the samples at room temperature (Rossi, 2019).

Various antihypertensive drugs alter renin activity (or concentration) and/or aldosterone secretion, influencing the ARR (Funder et al., 2016) (Table 2).

**TABLE 2:** Effect on antihypertensive drugs on aldosterone, renin, and aldosterone-to-renin ratio.

Drugs	Effect on aldosterone	Effect on renin	Effect on ARR	Interpretation when testing on drug
Beta-blockers	↓	↓↓	↑ (FP)	Increased ARR clinically not important (false-positive) if aldosterone low
Clonidine	↓	↓↓	↑ (FP)	Same as for beta-blockers
Methyldopa	↓	↓↓	↑ (FP)	Same as for beta-blockers
Calcium blockers (DHP)	↔↓	↔↑	↓ (FN)	Considered non-interfering in the 2020 Italian guidelines
Verapamil	↔	↔	↔	Considered non-interfering
ACEI	↓	↑↑	↓ (FN)	High renin does not exclude PA, testing must be repeated off-drug; low renin is strong predictor of PA
ARB	↓	↑↑	↓ (FN)	Same as for ACE inhibitors
Potassium-wasting diuretics	↔↑	↑↑	↓ (FN)	Considered prohibited during testing
MRA	↔/↑	↔/↑↑	↔/↓ (FN)	Previously considered prohibited during testing; based on the recent data may be continued (also during a confirmatory test and AVS), especially in patients with severe hypokalemia and/or poor BP control, and diagnosis of PA can be made in patients on MRA if aldosterone is high and renin low. However, if renin is not suppressed, then MRA should be discontinued for 4–6 weeks before retesting
Alpha-blockers	↔	↔	↔	Considered non-interfering
Moxonidine	↔	↔	↔	Single study in normotensives; considered non-interfering in the 2020 Italian guidelines
Hydralazine	↔	↔	↔	Rarely used nowadays; considered non-interfering

ACEI, angiotensin-converting enzyme inhibitors; ARB, angiotensin receptor blockers; ARR, aldosterone-to-renin ratio; AVS, adrenal venous sampling; BP, blood pressure; DHP, dihydropyridines; FN, false negatives; FP, false positives; MRA, mineralocorticoid receptor antagonist; PA, primary aldosteronism.

Beta-adrenergic blockers and central antagonists increase plasma aldosterone levels and decrease renin levels, thus increasing ARR and leading to false positive results (Bühler et al., 1972; Gordon et al., 1992; Manhem and Hökfelt, 1981). Newer studies also disclosed a high rate of false-positive ARR for PA with both DRC or PRA, ranging from 8 to 31% during beta-blocker treatment (Browne et al., 2016; Griffin et al., 2016). In contrast, moxonidine in normotensive, non-medicated male volunteers was not associated with a significant change in the ARR (Ahmed et al., 2017).

Diuretics stimulate renin more than aldosterone secretion, causing falsely negative ARR values (Rossi et al., 2020a), while dihydropyridine calcium antagonists lower ARR (Fiad et al., 1997).

Angiotensin-converting enzyme inhibitors (ACEI) and angiotensin II receptor blockers (ARB) increase renin and decrease PAC and, therefore, lower the ARR. In patients with PA, ACEI appear to be much less able to stimulate PRA than in those without PA, and are therefore unlikely to cause a false negative ARR (Lyons et al., 1983). The effects of ARB on aldosterone and PRA levels are similar to those of ACEI (Sica and Hollenberg, 1999).

The current guidelines state that a washout of all interfering antihypertensive medications before ARR testing is feasible in patients with mild hypertension but it is potentially problematic in others (Funder et al., 2016). Therefore, altering the antihypertensive treatment to medications that have a minimal effect on the ARR is suggested. Renin levels may rise modestly following commencement of verapamil (Guthrie et al., 1983) or hydralazine (Leenen et al., 1987), but rarely to an extent which significantly affects the ARR. Prazosin and doxazosin do not have a significant effect on ARR (Leenen et al., 1987; Oliveros-Palacios et al., 1991; Whitworth et al., 1987).

The effect of different drug therapies on ARR has rarely been systematically evaluated in prospective studies (Mulatero et al., 2002). Gallay et al. reported that high cut-off values of ARR above 100 (expressed in ng/dL over ng/mL/h) were identified in 15 among 90 patients with poorly controlled hypertension without discontinuation of their multidrug therapy regimen, but the performance of lower cut-off values was not provided (Gallay et al., 2001).

Seifarth et al. analyzed PAC and DRC in 37 normotensive controls, 144 hypertensive patients with essential hypertension and 19 patients with PA on single drug or combination therapy with beta-blockers, ACEI, ARB, calcium antagonists, spironolactone, or no treatment (Seifarth et al., 2002). In patients with essential hypertension, beta-blocker treatment led to a highly significant suppression of renin, whereas PAC was not significantly altered. This might lead to false-positive results when screening for PA. In ACEI-or ARB-treated patients with essential hypertension on monotherapy or combination therapy analyzed together (with exclusion of those receiving beta-blockers), PAC was decreased and renin levels were elevated, contributing to lower ARR. The alteration in PAC caused by ACEI and ARB was in the authors’ opinion unlikely to produce false-negative results in patients with APA because of autonomous aldosterone production. However, interference with milder forms of PA, such as bilateral adrenal hyperplasia, cannot be excluded. Calcium antagonists had no significant effect on PAC or DRC. Because of the small number of patients with known PA receiving antihypertensive drugs, significant differences in PAC, DRC and ARR between treated and untreated patients could not be identified. In patients with PA treated with spironolactone, renin reached very high levels. The authors concluded that calcium antagonists, and probably also ACEI and ARB alone or in combination, may be continued during screening for PA with ARR, in contrast to beta-blockers or spironolactone.

Mulatero et al. in their classical randomized study investigated the effects of therapy with atenolol, amlodipine, doxazosin, fosinopril, and irbesartan on the ARR in a group of 230 patients with suspected PA based on screening ARR >50 with PAC >10 ng/dl (Mulatero et al., 2002). Patients after 4 weeks of wash out were randomized to the monotherapy groups and observed for 2 months. PA was confirmed in 154 cases. The percent ARR change compared to the control was −17% ± 32 in patients taking amlodipine, + 62% ± 82 for atenolol, −5% ± 26 for doxazosin, −30% ± 24 for fosinopril, and −43% ± 27 for irbesartan. A false-negative ARR was obtained in 1.8% patients in the amlodipine group, and in 23.5% patients in irbesartan group, but the rate was not significant in the remaining groups. The authors concluded that doxazosin and fosinopril can be used in hypertensive patients who undergo ARR assessment for the diagnosis of PA. Amlodipine gave a very small percentage of false-negative diagnoses and beta-blockers can be responsible for an increased rate of false-positive ARRs.

A recently published prospective study performed in 42 consecutive patients with an unambiguous diagnosis of PA evaluated whether ARR values were affected by the MRA canrenone and/or by canrenone plus olmesartan treatment in patients with PA (Rossi et al., 2020c). Patients were treated for 1 month with canrenone, and for an additional month with canrenone plus olmesartan. Both drugs were up-titrated until blood pressure and potassium levels were controlled. Canrenone neither lowered plasma aldosterone nor increased renin and therefore the high ARR and true positive rate remained unaffected. The addition of ARB increased renin and slightly decreased aldosterone, which in turn reduced the ARR and increased the false negative rate. The authors concluded that canrenone did not preclude an accurate diagnosis in patients with PA, while the addition of the ARB only slightly raised the false negative rate.

The results discordant with the above were recently obtained in a retrospective cohort study in 146 patients with hypertension, including 91 with PA, who had plasma aldosterone and renin measurements before and after initiation of MRA treatment (Tezuka and Turcu, 2020). Initiation of MRA was associated with an increase in renin and aldosterone, and a decrease in ARR, irrespective of the MRA treatment duration and use of other antihypertensive medications. The authors concluded that MRA commonly reduce ARR and the proportion of positive PA screening results, and thus screening should be repeated off MRA when PA is suspected.

## Confirmatory Tests for Primary Aldosteronism

According to the Endocrine Society, confirmatory tests are considered mandatory for the definite diagnosis of PA (Funder et al., 2016). These recommendations suggest application of one or more confirmatory tests to validate or exclude PA. The most commonly used tests comprise the saline loading test, fludrocortisone suppression test and captopril challenge. So far, there is no clear evidence that any of these tests is optimal and has a diagnostic advantage over the others. As stated in the 2016 Endocrine Society guidelines, testing is not necessary and can be omitted in subjects with spontaneous hypokalemia, PRA <1.0 ng/mL/h (or DRC below the detection level) and accompanying PAC exceeding 20 ng/dl (550 pmol/L) (Funder et al., 2016).

Salt loading test is usually performed by either direct salt loading or intravenous salt infusion. During direct salt loading, the patient should receive approximately 6 g of salt daily (>200 mmol) for 3 days. Then, both aldosterone and sodium excretion should be assessed in a 24 h urine collection. A urinary aldosterone excretion <10 μg/24 h (28 nmol/d) indicates a low probability of PA, while a result >12 μg/24 h (>33 nmol/d) supports its diagnosis. The saline infusion test is usually performed in the morning in a patient remaining supine for 1 hour prior to testing. Then, over a 4 h period, 2 L of 0.9% saline are administered with careful blood pressure and heart rate monitoring. Blood samples for renin, aldosterone, cortisol and potassium determination are drawn at 0 and 4 h. A post-infusion PAC <5 ng/ml (140 pmol/L) decreases, while PAC >10 ng/ml (280 nmol/L) increases the probability of PA. It should be stressed that the saline infusion test may be not reliable due to failure to sufficiently differentiate between subjects with primary hypertension and normokalemic patients with PA (Schirpenbach et al., 2006).

Both the above tests should be performed under careful medical supervision. Due to significant sodium and fluid overload, these tests are not recommended in subjects with uncontrolled hypertension, left ventricular failure and renal insufficiency and marked hypokalemia.

During the fludrocortisone suppression test, the patient receives 0.1 mg of fludrocortisone every 6 h for 4 days. Additionally, potassium chloride supplementation and a high-sodium diet are necessary to maintain the appropriate plasma potassium concentration and ensure adequate urine sodium excretion. Plasma aldosterone and renin activity should be measured at 10 AM and cortisol at 7 and 10 AM on the last day of the test. The diagnosis of PA can be confirmed when the PAC of >6 ng/dl is accompanied by PRA <1 ng/mL/h and the cortisol concentration is lower at 10 AM than at 7 AM.

The captopril challenge test consists of administering 25–50 mg of captopril orally to the patient sitting or standing for 1 h. In patients with PA, PAC remains increased at suppressed PRA. The test is easily performed and safe for patients who are at risk due to compromised renal or cardiac function (Funder et al., 2016).

As confirmatory tests are performed after the initial diagnosis of PA including ARR measurement, the recommendations regarding the use of various antihypertensive drugs during testing are the same as before determination of ARR.

If it is possible based on the patient’s clinical status, i.e. blood pressure values, MRA should be withdrawn for at least 4 weeks while other antihypertensive drugs should be withdrawn for at least 2 weeks before testing. Replacement of other antihypertensive drugs with calcium antagonists or alpha-blockers may minimize interference with aldosterone and renin measurements. In one study, the effect of chronic antihypertensive medications on confirmatory testing results was investigated (Solar et al., 2012). The study enrolled subjects with suspected PA who underwent two consecutive oral plus intravenous sodium loading tests after prior division into two groups. In the first group, both tests were performed on guideline-recommended therapy with exclusion of drugs which interfere with the renin angiotensin system (Funder et al., 2008). In the second group, the first test was performed on chronic therapy but diuretics and MRA were withdrawn, while the second test was performed on the guideline-recommended therapy. Agreement in the interpretation of the two tests was found in 84% subjects in the first group and in 66% subjects in the second group. Additionally in the first group, all subjects with PAC ≥240 pmol/L still had nonsuppressible aldosterone during the next test. Similarly, in the second group all subjects with PAC ≥240 pmol/L on modified chronic therapy had nonsuppressible aldosterone during guideline-recommended therapy. The authors concluded than confirmatory testing performed while the patient is on chronic therapy without diuretics and MRA can confirm the diagnosis of PA, provided PAC remains markedly elevated at the end of saline infusion. The results of this study suggest that some types of antihypertensive therapy that are conventionally considered interfering may not have a significant effect on the results of confirmatory tests.

Importantly, some authors question the utility of confirmatory testing, instead suggesting proceeding directly to PA subtype determination by AVS if the patient is a candidate for surgery to remove APA. According to the Italian authors (Rossi et al., 2020a; Rossi et al., 2020b), the use of confirmatory tests remains controversial as they are based on the unproven premise that aldosterone secretion would be completely independent of renin and angiotensin II. These authors also note that studies supporting the use of confirmatory tests use did not comply with the Standards for Reporting of Diagnostic Accuracy (STARD) requirement for validation for diagnostic tests (Bossuyt et al., 2003). In the addition, the largest study that examined the captopril challenge test and fulfilled these requirements failed to show any diagnostic gain of this test over a carefully done ARR (Maiolino et al., 2017). Thus, it was postulated in the recent Italian guidelines that if consistently elevated ARR values are found during properly performed screening, and a “florid” PA phenotype is present (identified by PAC >15 ng/dl and DRC ≤2 mIU/L, with or without hypokalemia), the confirmatory tests are useless and one may proceed directly to subtyping if the patient is candidate for surgery (Rossi et al., 2020a; Rossi et al., 2020b). This approach is thus basically consistent with the one proposed in the 2016 Endocrine Society guidelines, i.e., to omit the confirmatory testing in patient with unambiguous PA phenotype, but the defining criteria vary between these guidelines.

## Discussion

According to the 2016 Endocrine Society guidelines, ARR remains the first line (screening) test for the evaluation for PA. If ARR is abnormal (elevated), a confirmatory test is needed, except for patients with spontaneous hypokalemia, plasma renin below the detection threshold and plasma aldosterone >20 ng/dl (Funder et al., 2016).

It is generally recommended to measure ARR after discontinuation of drugs that might affect renin and/or aldosterone. While it may be easily done in patients with mild hypertension, withdrawal of antihypertensive drugs may lead to suboptimal blood pressure control for several weeks in patients with more severe hypertension, with potential adverse consequences. The risks of modifying antihypertensive medication programs include hypertensive crisis, severe hypokalemia, atrial fibrillation, and heart failure. In one study, it was shown that withdrawal of the interfering drugs was possible in only half of patients, and 12% of them had a serious event necessitating hospital admission (Fischer et al., 2011).

Thus, an important question arises which drugs should be discontinued and which may be used for blood pressure control during the testing period.

Beta-blockers and central agonists (clonidine, methyldopa) decrease renin and increase aldosterone, thus artificially elevating ARR. Potassium-wasting and -sparing diuretics increase renin more than aldosterone, and thus artificially decrease ARR. Spironolactone was shown to significantly increase renin and reduce ARR in an older study (Seifarth et al., 2002), and the same was shown recently for MRA in a retrospective cohort study (Tezuka and Turcu, 2020), while in another recent prospective study in patients with an unambiguous diagnosis of PA (Rossi et al., 2020c), canrenone, the active metabolite of spironolactone neither lowered plasma aldosterone nor increased renin and thus did not affect high ARR in these patients. Calcium antagonists have generally little effect on ARR, but dihydropyridines tend to decrease ARR, and thus a false negative result may be obtained in mild PA (such as bilateral adrenal hyperplasia) (Nishikawa et al., 2011). In contrast, verapamil has a generally neutral effect on ARR (Funder et al., 2016), similarly to alpha-blockers and moxonidine (albeit tested in only one study in normotensives). ACEI and ARB reduce aldosterone and increase renin, thus decreasing ARR.

Overall, the conclusions from the studies that specifically evaluated whether ARR measurement and diagnostic interpretation are affected by ongoing treatment with various antihypertensive drug classes vary. Seifarth et al. (2002) concluded that beta-blockers led to false positive ARR, while calcium antagonists and possibly also ACEI and ARB could be continued during the testing. Mulatero et al. (2002) showed that atenolol increased ARR by on average 62%, while amlodipine, fosinopril, and irbesartan decreased ARR by on average 17, 30, and 43%, respectively, which led to a false-negative ARR result in nearly one fourth of patients receiving ARB, compared to only 1.8% of patients receiving calcium antagonist. Finally, doxazosin was also tested in the study by Mulatero et al. and was shown to have the least effect on ARR of all five evaluated antihypertensive drugs from various classes.

In summary, studies consistently show that diuretics artificially decrease ARR (risk of false-negative results), while beta-blockers and central agonists artificially elevate ARR, which may increase the rate of false-positive ARR results. Conclusions regarding the feasibility of measuring ARR on ACE/ARB vary, while calcium antagonists (particularly verapamil, but likely also dihydropyridines), alpha-blockers and moxonidine are not interfering much with ARR measurement and thus may be continued.

Regarding the feasibility of measuring ARR during MRA treatment, this issue seems more complex. The finding by the Rossi group that canrenone did not preclude an accurate diagnosis in patients with PA, is at variance with previous thinking and statements that MRA should be withdrawn before ARR measurement (Funder et al., 2016). However, the view that MRAs can be used during the screening for PA because they did not cause false negative ARR values during the screening (Rossi et al., 2020c), and also when PA patients undergo subtype differentiation, as these drugs did not preclude the diagnosis of unilateral PA by AVS (Haase et al., 2014; Nanba et al., 2019), was later upheld in two Italian guideline documents (Rossi et al., 2020a; Rossi et al., 2020b), despite findings of other studies, both earlier and recent, that suggest otherwise (Seifarth et al., 2002; Tezuka and Turcu, 2020). How to reconcile these apparently contradictory findings? MRA prevent aldosterone from activating the receptor, which leads to sodium loss and reduction of plasma volume, and this should subsequently reduce PRA, thus limiting the utility of the PAC/PRA ratio. One possible explanation might be differences in patient characteristics between these studies, as Rossi et al. (2020c) studied patients with florid PA phenotype and very high ARR, while other studies evaluated more general population of hypertensive patients screened for PA. Indeed, patients with the study by Rossi et al. (2020c) had low renin before initiation and during administration of canrenone, which is consistent with the previous stipulation in the 2016 Endocrine Society guidelines (along with the general recommendation to withdraw/not initiate MRA before the testing) that even MRA treatment may be continued in specific situations during testing for PA, for example in some patients with severe PA. In these settings, testing for PA may be pursued on as long as renin is shown to be suppressed.

Overall, the above study findings are largely reflected by the recommendations regarding drug withdrawal for ARR measurement included in the 2016 Endocrine Society guidelines (except for the 2020 Rossi study which was published later, and could only be reflected in the most recent Italian guidelines).

According to the 2016 Endocrine Society guidelines, aldosterone antagonists, other potassium-sparing diuretics, and potassium-wasting diuretics markedly affect ARR and should be withdrawn for at least 4 weeks. Of note, some other older guidelines suggested withdrawal of these drugs for a longer time, even 6 weeks or longer (Nishikawa et al., 2011). If ARR is not diagnostic after discontinuation of the above drugs and hypertension can be controlled with noninterfering medications, the 2016 Endocrine Society guidelines suggest that other medications such as beta-blockers, ACEI, ARB and dihydropyridine calcium antagonists should be withdrawn for at least 2 weeks. According to these authors, the least interfering drugs are verapamil, alpha-blockers, and hydralazine (which is currently rarely used for the treatment of hypertension), and thus these should be preferentially used when drugs are needed to control hypertension in patients evaluated for PA.

The authors of the 2020 Italian guidelines (Rossi et al., 2020a) agree that diuretics, ACEI, ARB, and beta-blockers must be stopped before sample collection. They also stated that in their experience, renin can be falsely high with ACEI and ARB, and falsely low with beta-blockers, even three weeks after stopping these drugs. Therefore, they recommend withdrawal of these agents for at least 4 weeks. According to the 2020 Italian guidelines, long-acting dihydropyridine calcium antagonists, alpha-blockers (doxazosin) and/or moxonidine should be used to control hypertension for 4–6 weeks before measuring the ARR because these drugs minimally affect renin and PAC. Non-dihydropyridine calcium antagonists, verapamil and diltiazem, are alternatives to dihydropyridines, particularly in patients who do not tolerate the dihydropyridine calcium antagonists, based on widespread clinical experience that has shown that verapamil slow release and diltiazem do not decrease the sensitivity of PA screening.

Importantly, as noted above, the two recently published Italian guideline documents (Rossi et al., 2020a; Rossi et al., 2020b) allow use of MRA when screening for PA by ARR measurement, and also during PA subtype differentiation by AVS. The most obvious candidates for ARR measurement on continued MRA treatment are patients with severe hypokalemia and/or poor blood pressure control on alternative treatment. However, as stated in these guidelines (Rossi et al., 2020a), the diagnosis of PA can be made in patients on MRA if PAC is high and renin low.

**FIGURE 2:**
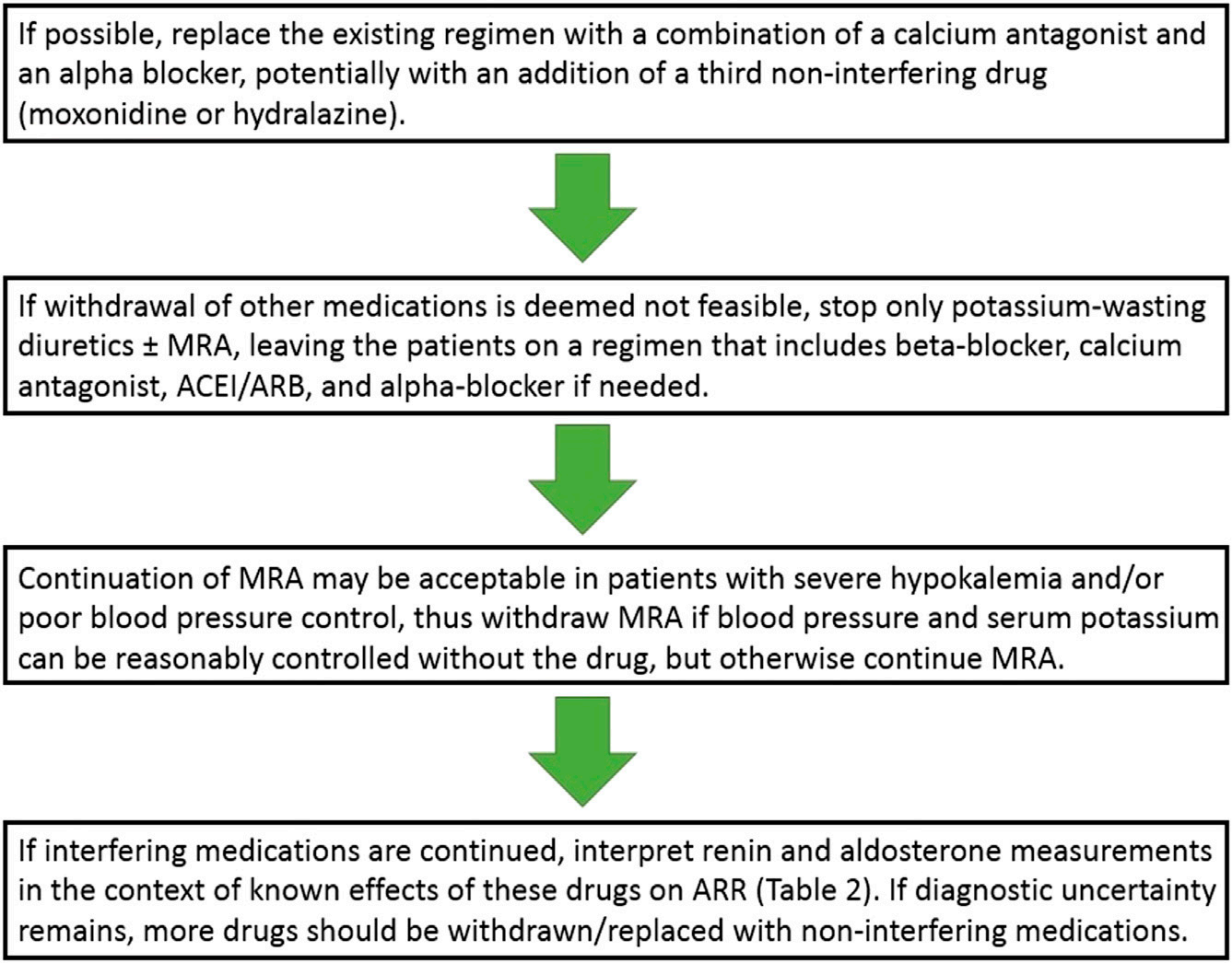
Approach to antihypertensive drug regimen modifications when screening for primary aldosteronism. ACEI, angiotensin-converting enzyme inhibitors; ARB, angiotensin receptor blockers; ARR, aldosterone-to-renin ratio; MRA, mineralocorticoid receptor antagonist.

Our recommendation for the extent and order of drug withdrawal for ARR measurement would be thus as follows (Figure 2).

If hypertension is not very severe and it may be expected to be reasonably controlled on two or three medications, an attempt could be made to replace the existing regimen with a combination of a calcium antagonist and an alpha blocker, potentially with an addition of a third non-interfering drug (moxonidine or hydralazine). Regarding the choice of a calcium antagonist, verapamil could be preferentially used (Funder et al., 2016) but a dihydropyridine is a reasonable alternative (Rossi et al., 2020a). Of note, however, combining a dihydropyridine with doxazosin as the sole two-drug combination is frequently associated with lower limb edema and patients should be warned accordingly to increase treatment compliance.

If for some reasons such an antihypertensive drug regimen switch is deemed not feasible (e.g., due to severity of hypertension, a recent cardiovascular event, or compelling indications for continuing some drug classes), only diuretics should be stopped (either potassium-wasting or sparing, including MRA if possible–but not necessarily in all patients, see below) for 4 weeks, and the patient may be left on a regimen that includes beta-blocker, calcium antagonist, ACEI/ARB, and alpha-blocker if needed.

As noted above, recent data indicate that MRA may not need to be discontinued before testing for PA, but we believe, as noted in the recent Italian guidelines (Rossi et al., 2020a; Rossi et al., 2020b), that continuing MRA would be most useful primarily in specific patient subsets, such as patients with severe hypokalemia and/or severe/resistant hypertension, so the prudent approach would be to withdraw MRA if blood pressure and serum potassium can be reasonably controlled without the drug, but otherwise continue MRA.

Following these antihypertensive drug regimen modifications, ARR is measured but renin and aldosterone measurements should be interpreted in the context of known effects of these drugs on ARR (Table 2). Only if diagnostic uncertainty remains, more drugs should be withdrawn/replaced with non-interfering medications for a period as short as 2 weeks before the testing. Furthermore, as noted below, if these drug modifications/withdrawals seem potentially risky but the decision is made to proceed with them anyway, it may be not desirable to expose the patient to these alterations more than once, and one should consider performing ARR and a confirmatory test at the same occasion.

When this alternative approach to perform ARR measurement on interfering antihypertensive drugs is pursued, the renin and aldosterone measurements must be interpreted taking into account the known effects of these drugs on ARR. In these settings, it is particularly important to look not only at the calculated ARR value, but also consider renin and aldosterone measurements separately. In particular, if ARR is elevated but this elevation is solely due to low renin with low normal aldosterone, a false-positive result is likely. For that reason, some authors recommend that elevated ARR value should be interpreted as abnormal only in conjunction with a certain threshold plasma aldosterone level. However, the threshold of PAC >15 nd/dl proposed by some authors (Funder et al., 2016) may result in false-negative ARR, particularly in patients with bilateral adrenal hyperplasia as opposed to APA. Thus, some investigators proceed with a diagnostic workup for PA in all patients with elevated ARR unless the PAC is below the level used to define normal suppression during confirmatory suppression testing (e.g., 5 ng/dl), which results in more false positive but fewer false negative ARR results (Funder et al., 2016).

If necessary, most antihypertensive medications can be continued. For example, although beta-blockers lower renin and raise ARR, the increased ARR is not clinically important in this setting because of the low PAC (<10 ng/dl) in patients without PA (Ahmed et al., 2010).

ACEI and ARB elevate renin and reduce PAC, leading to a risk of a false-negative ARR. Therefore, a high renin (PRA >1 ng/mL/h or high DRC) in a patient treated with ACEI/ARB does not exclude PA. On the other hand, low renin (PRA <1 ng/mL/h or low DRC) in a patient taking one of these drugs is a strong predictor for PA. Hence, discontinuation of these medications is often not necessary but repeat assays must be performed off-treatment if renin is found to be high during on-drug testing (Nishikawa et al., 2011).

Similar interpretation applies to testing on MRA. In specific situations, for example in some patients with severe PA, as discussed above, aldosterone and renin may be measured in patients treated with MRA, and if renin is suppressed, these medications are not interfering, particularly if used in low doses). Hence, if renin is found to be suppressed, further testing (including confirmatory testing and AVS) can be performed without discontinuing MRA. However, if renin is not suppressed, then MRA therapy should be discontinued for 4–6 weeks before retesting.

Our recommendations regarding antihypertensive drug withdrawal before a confirmatory test would be generally consistent with the above recommendations regarding drug treatment modifications before ARR measurement. The authors of the 2016 Endocrine Society guidelines recommended that clinicians utilize the pharmacological agents with minimal or no effects on the renin-angiotensin-aldosterone system, and should avoid medications known to stimulate renin during confirmatory testing because these prevent the suppression of aldosterone (a false positive result). Thus, it would be best to limit the antihypertensive treatment to calcium antagonists (particularly verapamil), alpha-blockers, moxonidine and/or hydralazine, while diuretics and ACEI/ARB should be avoided. In the light of the recent data, MRA could be used if necessary, as long as renin is shown to be suppressed.

For practical reasons, if ARR is first performed on some interfering medications and then a decision is made to withdraw these medications anyway due to difficult interpretation of the ARR measurement, it might be reasonable to perform the repeated ARR measurement along with a confirmatory test, such as intravenous saline loading (and/or AVS, depending on whether one is still relying on confirmatory testing or agrees with the approach advocated by Rossi et al., i.e., to omit confirmatory testing and proceed directly from ARR measurement to AVS). This would require withdrawal of antihypertensive medications only once and/or for a shorter period, in contrast to a situation, e.g., where ARR is re-measured first on an outpatient basis and only later a decision is made to perform further inpatient testing (a confirmatory test and/or AVS).

***Research gaps and potential developments in the field.*** The effect of antihypertensive medications on ARR was evaluated in various populations including healthy volunteers, patients preselected according to ARR or in patients with confirmed PA only. The medication regimens, e.g., one or two drug combinations, as well as specific drugs did not necessarily reflect well the usual/current clinical practices in the management of hypertension. In addition, the cut-off values for positive ARR were higher than commonly accepted in the clinical practice. Thus, more studies, including prospective and randomized ones, are needed, as well as studies in larger patient populations. In particular, more studies are needed that would be based on lower cut-off values for positive ARR and that would evaluate the effects of antihypertensive drugs in various patient subsets, e.g., general population of patients evaluated for PA, in contrast to patient populations with an established diagnosis of PA or even “florid” PA phenotype in which many studies discussed in this review were performed.

Discordant data are available regarding the reproducibility of ARR (Yozamp et al., 2021; Rossi et al., 2010). Variation in ARR may be related to varying testing conditions, including the effect of various antihypertensive drug classes used during ARR measurement. Hence, more data on the effect of various antihypertensive drug classes on the reproducibility of ARR would be welcome.

Based on the recent literature, there are discordant/evolving conclusions regarding the feasibility of MRA use during testing (Rossi et al., 2020c), as opposed to a significant interfering effect of MRA and the need to withdraw these medications (Funder et al., 2016; Tezuka and Turcu, 2020). Again, this may be related to the fact that these discrepant results were obtained in various patient populations, e.g., “florid” PA phenotype vs. more general populations of hypertensive patients screened for PA.

A relatively “novel” approach of bypassing a confirmatory test and proceeding directly to AVS (Rossi et al., 2020a; Rossi et al., 2020b) also requires more data on the effect of various antihypertensive drug classes on the diagnosis of PA based on ARR measurements only. There are also ongoing attempts to develop prediction scores to avoid confirmatory testing and/or AVS (Rossi et al., 2020a; Burrello et al., 2020). If these attempts prove successful, information on the effect of hypertensive drugs in patients evaluated using such scores will be needed.

## Conclusion

Testing for PA should be ideally performed off-drugs that interfere with the renin-angiotensin-aldosterone axis. However, the need to withdraw these drugs and/or modify the treatment accordingly for several weeks prolongs the overall diagnostic process, and the feasibility of testing in patients with more severe/resistant hypertension, very high cardiovascular risk, or severe hypokalemia is limited due the risks related to suboptimal control of blood pressure and serum potassium level in the evaluation period. On the other hand, testing while on interfering antihypertensive medications creates issues with the interpretation of renin and aldosterone measurements for the purpose of diagnosing PA due to the effect of these medications on the measured parameters. Our literature review indicates that the least interfering drugs are calcium antagonists, alpha-blockers, hydralazine, and possibly moxonidine. If necessary, the testing may also be attempted during treatment with beta-blockers, ACEI, and ARB but renin and aldosterone measurements must be interpreted in the context of known effects of these drugs on these parameters. Views are evolving on the feasibility of testing during treatment with MRA, as these drugs are now increasingly considered acceptable in specific patient subsets, particularly in those with severe hypokalemia and/or poor blood pressure control on alternative treatment.
